# Puzzled by dysfunctional breathing disorder(s)? Consider the Bayesian brain hypothesis!

**DOI:** 10.3389/fnins.2023.1270556

**Published:** 2023-10-09

**Authors:** Claudine Peiffer

**Affiliations:** Dyspnea Clinic, Department of Physiology, University Children Hospital Robert Debré (AP-HP), Paris, France

**Keywords:** dysfunctional breathing disorder, Bayesian brain hypothesis, symptom perception, dyspnea, predictive coding

## Abstract

There is currently growing clinical concern regarding dysfunctional breathing disorder(s) (DBD), an umbrella term for a set of multidimensional clinical conditions that are characterized by altered breathing pattern associated with a variety of intermittent or chronic symptoms, notably dyspnea, in the absence or in excess of, organic disease. However, several aspects of DBD remain poorly understood and/or open to debate, especially the inconsistent relationship between the array of experienced symptoms and their supposedly underlying mechanisms. This may be partly due to a more general problem, i.e., the prevailing way we conceptualize symptoms. In the present article, after a brief review of the different aspects of DBD from the current perspective, I submit a call for considering DBD under the innovating perspective of the Bayesian brain hypothesis, i.e., a potent and novel model that fundamentally changes our views on symptom perception.

## 1. Introduction

### 1.1. Dysfunctional breathing disorder from the current perspective: what is well established and what remains fuzzy?

There is currently growing interest and awareness concerning dysfunctional breathing disorder(s) (DBD), an umbrella term (used throughout this article) for a set of poorly distinguishable clinical conditions including the most emblematic and anciently known hyperventilation syndrome. Either in isolation or in combination with other diseases, notably asthma (Thomas et al., 2001, 2005), DBD affects in a variable proportion (between 5 and 35%) both adults (Thomas et al., 2001; Agache et al., 2012; Connett and Thomas, 2018) and children (D'Alba et al., 2015; Connett and Thomas, 2018; Vahlkvist et al., 2023) in a variable proportion (between 5 and 35%), with a highly negative impact on health-related quality of life (Chenivesse et al., 2014). DBD is consensually considered as a condition encompassing one or several forms of altered breathing pattern (dysfunctional breathing) associated with an array of intermittent or chronic symptoms that may be respiratory, notably dyspnea, and/or non-respiratory in the absence or in excess of, organic disease (*reviewed in*: Barker and Everard, 2015; Boulding et al., 2016; Vidotto et al., 2019; Barker et al., 2020; Denton et al., 2022). Consequently, these different symptoms, including dyspnea, are commonly referred to as “functional,” “disproportional,” “poorly” or even “unexplained,” and therefore, DBD may be considered as part of the large nebula of the so-called functional syndromes (scFS) in the absence of a generally accepted term for this multiform nebula of syndromes (Creed et al., 2010). DBD encompasses a large variety of different, mostly co-existing types of dysfunctional breathing (DB) types during exercise and mostly at rest, e.g., hyperventilation in excess of metabolic demand, irregular breathing with or without frequent sighing, thoracic dominant breathing and exercise-induced laryngeal obstruction (Barker and Everard, 2015; Boulding et al., 2016; Vidotto et al., 2019; Barker et al., 2020; Ionescu et al., 2021) that may be associated to changes in breathing movement patterns, e.g., thoracic dominant and paradoxical breathing (Van Dixhoorn, 2004; Depiazzi and Everard, 2016). DB is characterized by a chaotic behavior (Bokov et al., 2016; Bansal et al., 2018) and a decreased adaptability to various requirements, i.e., mainly physiological ones (e.g., metabolic needs of the body) but also cognitive and/or emotional ones (Courtney, 2016; Vlemincx, 2023). Typically, clinical expressions of DBD are highly variable across and even with-in subjects in terms of dyspnea intensity, as well as of severity, nature and number of the before-mentioned types of DB and of the numerous non-respiratory symptoms (e.g., vertigo, light headedness, paraesthesia, chest pain, tachycardia, tremor, headache, muscle cramping, stiffness around the mouth, bloated stomach fatigue, and sleep disturbances). From the currently prevailing perspective, there remain several concerns regarding thorough characterization of DBD. Indeed, given the striking absence a gold standard definition and consequently, of reliable diagnostic tests, “diagnosis” of DBD is actually at best a fair estimation or suspicion reflecting the doctor’s gut feeling, itself based on a bundle of arguments taking into account the above-mentioned characteristics. Unfortunately, however, neither dyspnea nor DB or any of the numerous non-respiratory symptoms of DBD or the inconstantly associated pathophysiological changes (especially the most emblematic one, namely hypocapnia) are truly specific of DBD. Indeed, all may be present in each of the variety of clinical conditions that may coexist or overlap with, and thus, be hardly distinguishable from DBD, e.g., affective disorders notably, panic disorder (Roth, 2005; Sikter et al., 2007), other scFS, e.g., fibromyalgia and low back pain (Bogaerts et al., 2007; Ramakers et al., 2022), but also organic disease, especially asthma (Thomas et al., 2001; Connett and Thomas, 2018) and long COVID (Bouteleux et al., 2021; Motiejunaite et al., 2021; Frésard et al., 2022) and even some of them, e.g., DB, occasionally in healthy subjects (Vlemincx, 2023). Furthermore, as several physiological parameters, notably breathing patterns, present a broad and continuous range of possible values across subjects (including healthy subjects) as well as high intra-subject variability, it is often difficult to determine a clear-cut limit between “normal” and “DBD-related values” (Han et al., 1997). In addition, while the strong bilateral relationship between DB and symptoms of DBD and emotion, especially anxiety and stress, is well recognized (Han et al., 1997, 2004; Gilbert, 1998; Courtney, 2016), the underlying mechanisms are not yet fully understood. The greatest challenges, regarding DBD (as scFS in general), lies however in the fact that the before-mentioned symptoms have a weak, variable but mostly, no relationship at all with the supposedly underlying physiological changes (Stoop et al., 1986; Hornsveld et al., 1996), and that symptom reports in DBD patients may not be very helpful because of an imprecise, sometimes even unreliable characterization of the symptoms. Consequently, it is often difficult to disentangle the “functional” from the “organic” origin of these symptoms, i.e., a concern of many physicians referring patients for suspected DBD, based on the still prevailing and mostly implicit assumption that “organic” and “functional” symptoms are different in nature, the first being robust because related to a measurable and potentially treatable cause and the second, as being principally “in the head” and thus predominantly, a psychological issue. Yet, as suggested by highly convincing recent work (*reviewed in*: Van den Bergh et al., 2017), some of the before-mentioned concerns regarding DBD may actually correspond to a more wide-ranging conceptual issue, namely our current implicit view of symptom perception in general, i.e., considering symptoms as resulting from perception of physiological changes within the body via a direct bottom-up sensory input. Indeed, during the latest decades, symptom perception has been put in broader and more complex frameworks, notably by an alternative ground-braking model, the Bayesian brain hypothesis. Paradoxically however, these new insights are mostly overlooked in daily clinical practice. In the present article, I try to show why and how the Bayesian brain hypothesis appears to be an ideal model to explain the many different aspects of DBD that remained hitherto obscure and difficult to explain.

## 2. Dysfunctional breathing disorder from the innovative perspective of the Bayesian brain hypothesis

### 2.1. General presentation of the Bayesian brain hypothesis (where one can already guess its relevance for DBD)

The fundamental principle of the Bayesian brain hypothesis (BBH) is based on Bayes’ theorem. The latter, named after Thomas Bayes, a eighteenth century British mathematician, allows to determine the posterior probability (posterior) of a hypothesis given prior beliefs about its probability (prior) and the likelihood of relevant associated data, by an operation called Bayesian inference. The conceptual framework of the BBH further relies on the still-relevant hypothesis of von Helmholtz who first submitted in the nineteenth century that perception is an unconscious inference of the causes of sensation (Helmholtz, 1866), and includes the “free energy principal” (see below) as well as relevant theoretical input from recent hypotheses and models of interoception (Barrett and Simmons, 2015). Since a few decades, there is increasing body of evidence from research in neuroscience, notably the seminal work of Friston, that Bayesian inference is a fundamental characteristic of brain function (Rao and Ballard, 1999; Friston, 2003, 2005, 2010; Ma et al., 2006; Clark, 2013) (referred to as Bayesian predictive coding (BPC) throughout this article). The BBH has been most extensively assessed and verified in the field of sensory perception, particularly for vision (Rao and Ballard, 1999; Murray et al., 2002) and audition (Todorovic et al., 2011) and subsequently, with mutual conceptual input, for interoception (Seth et al., 2011, reviewed in Van den Bergh et al., 2017) and symptom perception (Büchel et al., 2014; *reviewed in*: Van den Bergh et al., 2017; Henningsen et al., 2018) including dyspnea (Faull et al., 2017, 2018; Marlow et al., 2019).

**Figure 1:**
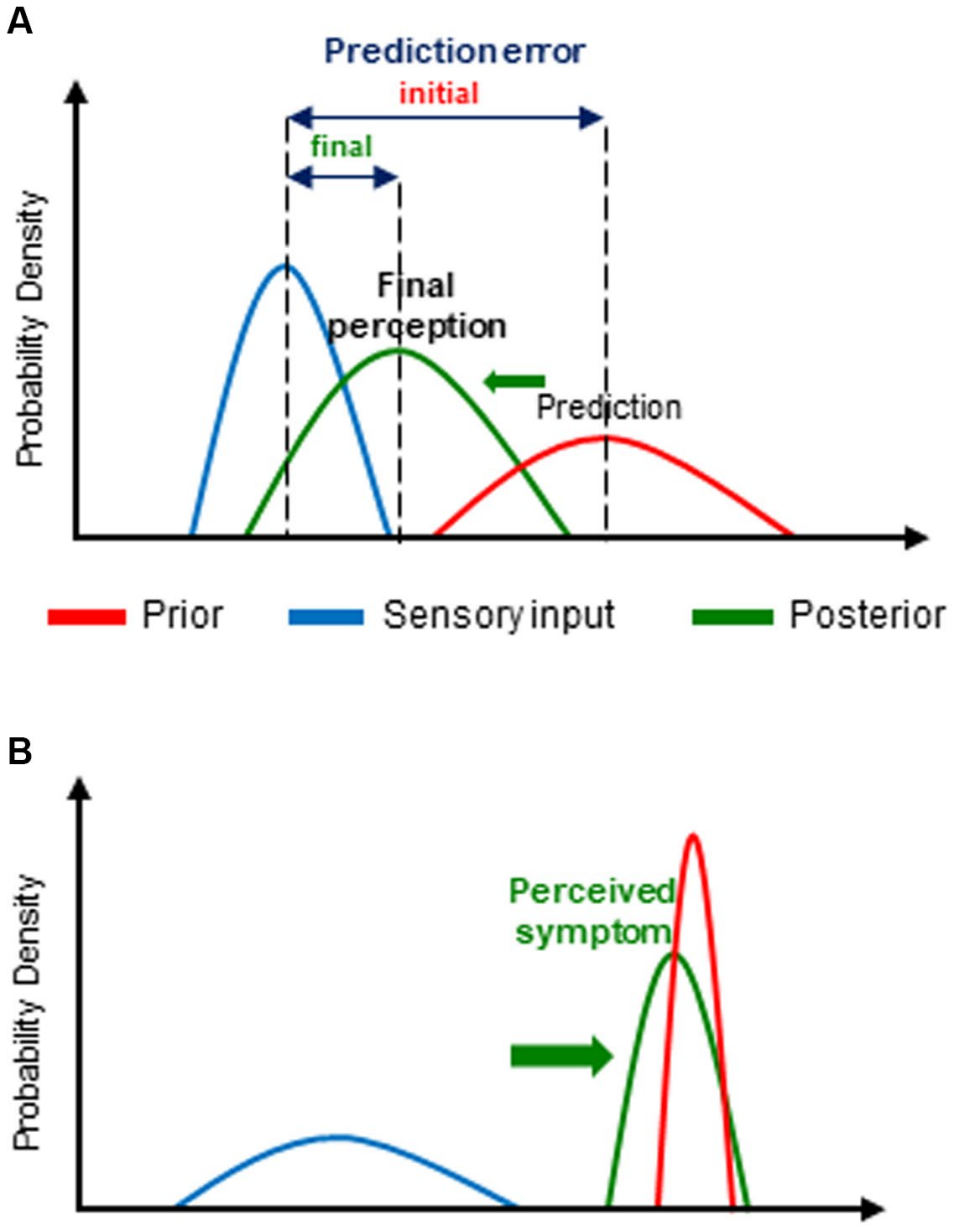
Bayesian predictive coding (BPC): **(A)** general principles of the model and **(B)** its application to dysfunctional breathing disorder(s) (DBD). **(A)** For the purpose of prediction error minimization, the initial prediction or expectation (prior) is updated in the light of new incoming information (sensory input). The resulting updated posterior belief (posterior) (and thereby, the corresponding conscious perception) is crucially determined by the relative weight, i.e., the precision, of the prior and of sensory input. Thus, the posterior is predominantly influenced by biased toward (→) the one of its 2 determinants with the highest precision (i.e., the narrowest distribution of its expected values). Each parameter is represented by its corresponding probability distribution. **(B)** In the case of DBD, the prior (prediction) is mostly abnormally precise and/or the sensory (physiological) input imprecise. Consequently, the posterior, i.e., the actually perceived symptom, is predominantly determined by the prior (that, moreover, is often erroneous).

This innovative hypothesis submits that, rather than being a neutral receptor of sensory input from the inner and/or outer world, to which it has no direct access, the brain is actually an active inference machine that generates unconscious predictions about the most likely causes of that sensory input (priors) which are compared with actual incoming sensory input. Priors are based on innate homeostatic values but predominantly, on expectation and beliefs themselves grounded on past experience and associative learning and on contextual cues. In the case of difference (mismatch) between the prior (predicted sensory input) and the input actually received, a prediction error (an error signal of unexpected sensory input or surprise) is generated. The latter leads to an update of the prior by improving prediction and thereby, to the formation of a so-called posterior that determines the final conscious perception, and in turn, will be the new prior for a subsequent sensory event (Figure 1A). This update consists of minimizing as much as possible the corresponding prediction error either by modifying the prior (by changing “expectations”) or by the way it samples afferent sensory information (Friston, 2005; Friston et al., 2006; Barrett and Simmons, 2015). Maximal reduction of prediction errors (i.e., sensory surprise) is indeed a fundamental characteristic of brain function (Friston, 2010, 2013) based on the “free energy principle,” a basic property of biological (self-organizing) systems, i.e., resistance against the tendency to entropy (disorder) (Friston et al., 2006; Feldman and Friston, 2010). Furthermore, the probabilistic nature of all the different components of the BPC process allows the brain to deal with uncertainty, an important feature of neural processing, in this case, uncertainty regarding prediction of sensory input by the prior, the sensory input itself (i.e., its signal to noise ratio) as well as associated prediction errors. The highest precision (highest weight or lowest uncertainty) is associated with the narrowest distribution of the probability of occurrence (likelihood) of a range of possible values of each of these variables. Precision and expected precision of the latter have a major impact on the final conscious perception (Friston, 2005). Indeed, the final posterior belief is biased toward the one of its determinants (prior belief and actual sensory input) with the highest precision (Figure 1A).

Thus, each given sensory input may generate a great number of different posterior beliefs, and ultimately, conscious perceptions, according to the different combinations of the relative precisions of the prior and the sensory input. Furthermore, the precision of the prior and of sensory input and thereby, their relative contribution to the posterior, are highly dependent on both contextual and individual factors which essentially impacts the way how the final perception is consciously experienced (Hohwy, 2012; Kanai et al., 2015; Van den Bergh et al., 2017; Friston, 2018). Contextual factors and cues include attention as well as conscious personal expectations and beliefs, themselves related to personal history as well as to the cultural and social backgrounds, whereas individual factors include personality traits, especially negative affect, gender and genetic factors (Edwards et al., 2012; Van den Bergh et al., 2017). Thus, for the crucial formation of the final perceptual construct, the BPC process involves also conscious high-ordered cognitive processes including interpretations (attribution of significance, hedonic and affective tone to sensory input) as well as expectation and beliefs involving associative learning (Van den Bergh et al., 1995, 1997, 2002) all of them also contributing to the unconscious prior formation. It is also noteworthy that, expected precision of sensory input significantly influences the importance and signification attributed to prediction errors (Van den Bergh et al., 2017) and prediction errors are modulated by predictions of their precision (Van den Bergh et al., 2017; Friston, 2018).

Moreover, according to a further important concept of the BPC model, the corresponding neuro-cognitive process is hierarchically organized, i.e., it takes place within and between multiple hierarchically structured and interacting cortical levels (Felleman and Van Essen, 1991; Mumford, 1992; Rao and Ballard, 1999).

This consists of a main (first-order) continuous, bi-directional flow of information through these levels running from lower to higher areas and backward from higher to lower of information through these levels, i.e., ascending (bottom-up) prediction errors related to sensory input and descending (top-down) predictions (priors) that are progressively updated. At each of these hierarchical levels, the prior is compared to sensory input with formation of a prediction error that is send to the level above to form a new posterior that will constitute the updated prior for the level below. The basic properties of incoming information are processed at lower hierarchical levels and its more complex and abstract aspects at the highest ones. Throughout this hierarchical process up to the highest level of integration (final estimate of the brain), priors are progressively refined as to obtain the most reliable prediction (optimal guess) of sensory input. In this respect, it is important to emphasize that the information actually conveyed by this ascending flow through the different hierarchical levels is the prediction error related to sensory input rather than the whole sensory information. i.e.; only its unpredictable part is computed since the remaining sensory information is already contained in the prediction. Thereby, the brain avoids unnecessary computational work with redundant information as well as neural signaling delay, which in turn, allows adaptive behavior and potentially life-saving anticipation (Gregory, 1980; Kveraga et al., 2007). Furthermore, the BPC model submits the presence of an additional (second-order) bi-directional flow of information dealing with the precision of prediction errors and of lateral connections within each hierarchical level (Kanai et al., 2015). Interestingly, it has also been shown that the hierarchical cytoarchitectonic structure as well as the intra- and inter-cortical connectivity of the brain are consistent with the BPC model (Bastos et al., 2012; Barrett and Simmons, 2015) which has been most extensively studied in the context of interoception (Barrett and Simmons, 2015). Thus, most cortical areas are composed of functional units and layers (or laminae) and each of the different components of BPC processing, i.e., priors, prediction errors and precision correspond to the activity of specific cells in terms of anatomical type and localization. Priors are encoded by superficial pyramidal cells located in granular cortices (predominantly the primary sensory cortex and posterior insula) whereas prediction errors are computed by deep pyramidal cells located in agranular cortices (predominantly in the anterior insula, the anterior and mid-cingulate cortex and posterior ventromedial prefrontal cortex) and sent back to the prior generation units of the level above (Friston, 2005; Shipp et al., 2013; Barrett and Simmons, 2015; Geuter et al., 2017). In addition, efferent copies of predictions are sent from the agranular cortex to multiple sensory systems across the brain to build up the ultimate conscious perception. Interestingly, in the context of proprioception, agranular visceromotor regions (where priors are generated) are considered to be relatively insensitive to prediction errors (Barrett and Simmons, 2015; Khalsa et al., 2018) thus highlighting again the predominant role of prediction in the final percept resulting from the BPC process. Likewise, precision corresponds to the activity of specific cells that modulate—mostly increase—synaptic gain (i.e., post-synaptic responsiveness) of cells encoding predictions and prediction errors (Friston, 2008; Barrett and Simmons, 2015). In functional motor and sensory symptoms, this increased gain has been shown to be related to misdirected attention from higher-level.

Finally, and most importantly, the BBH provides a highly innovating explanatory framework for the underlying mechanisms of a number of clinical conditions, i.e., basically, an alteration of the BPC process regarding precision weights of priors relative to sensory input and metacognitive interpretation of this input (Edwards et al., 2012; Adams et al., 2013; Van den Bergh et al., 2017). This explanatory model has been applied to neuropsychiatric disorders that are characterized by, or associated with abnormal concepts (delusion) (Schmack et al., 2013) or/and sensations, e.g., hallucinations and illusions and/or movements (Edwards et al., 2012; Adams et al., 2013; Notredame et al., 2014) and most interestingly, to scFS (*reviewed in*: Van den Bergh et al., 2017) or equivalent (Edwards et al., 2012).

### 2.2. Why and how does the Bayesian brain hypothesis change our way to consider DBD?

By creating several fundamental paradigm shifts, the BBH contribute substantially to a renewed insight into DBD due to major conceptual changes of our way to consider brain function and symptoms. The far most ground-braking of these paradigm shifts is that, according to the key concept of the BBH, namely active BPC, we do not see the world as it is but as we guess or expect it to be, that, by the way, is considered as a clever and adaptive way of brain functioning (Gregory, 1980; Kveraga et al., 2007). This predominant role of prediction in the BPC process moreover blurs the boundaries between prediction of perception and actual perception and consequently, conscious perceptions and symptoms may be regarded as “controlled hallucinations” (Clark, 2013; Seth, 2020) and, more specifically, led to the attractive hypothesis that scFS may be considered as a “somatovisceral illusions” (Van den Bergh et al., 2017). Above all, it appears that every consciously experienced perception, including symptoms, is actually a complex cognitive construct primarily based on top-down evolutive, potentially erroneous, predictions rather than being the mere result of a straightforward bottom-up sensory input in a one-to-one relationship. It also follows—and this is a further crucial BBH-related paradigm shift—that all symptoms induce the same conscious subjective experience of “trueness” whichever their degree of coupling to sensory input (in this case related to physiological function or dysfunction, hereafter referred to as physiological input). Most importantly, the BBH model—including similar theoretical frameworks that are more specifically focused on interoception and symptom perception (Van den Bergh et al., 2017; Henningsen et al., 2018; Paulus et al., 2019) provide an innovative and powerful explanatory framework for the underlying mechanisms of the scFS including DBD. Indeed, as previously mentioned, and further discussed below, DBD, can be reasonably considered as being part of the scFS nebula. Thus, for the symptom formation and perception of all the corresponding clinical conditions, the BBH submits a unifying pattern of altered BPC process. The latter consists basically of erroneous predictive coding, i.e., false inferences, that are mainly related to abnormally precise and mostly erroneous priors but also to, and interacting with imprecise physiological input both leading to a predominant influence of prior beliefs upon final perception (Figure 1B). These abnormal prior beliefs, are predominantly related to altered health beliefs involving several high-ordered conscious cognitive processes such as expectation of incoming health problems and ultimately, erroneous attribution of threatening health problem as the most likely cause of—sometimes minor—changes in physiological input. Interestingly, the relative contribution of priors, and consequently, that of contextual cues (leading to learned associations with symptoms) tends to increase over time, typically in long-lasting symptoms as scFS and presumably DBD, and may thereby explain their progressive decoupling from initial physiological input and potentially contribute to the perpetuation of these clinical conditions (de Peuter et al., 2005). Furthermore, as shown by previous studies, scFS is frequently associated with personality traits such as negative affect, sensitivity to threat, body-focused attention and heightened concern about the body and health, catastrophizing interpretation of minor bodily complaints, decreased tolerance to uncertainty and to unpleasantness, context rigidity (Rief et al., 1998; Van den Bergh et al., 2017; Paulus et al., 2019). Many of these personality traits are also present in subjects prone to anxiety and/or negative affect but otherwise healthy, that are moreover high symptom reporters (Bogaerts et al., 2015) The latter have also been shown to have greater vulnerability for scFS and threat that is itself linked to anxiety (Van den Bergh et al., 2017). Thus, the association between these different characteristics may uncover the link between anxiety and scFS and thus contribute to shed some new light into the commonly observed yet poorly understood relationship between anxiety and scFS and more specifically, DBD. Indeed, it has been shown that severe trait and state anxiety is more frequent in “functional” than “organic” respiratory diseases in children, especially in girls (Nevoia et al., 2022). In this regard, valuable insight also comes from two recent functional brain imaging studies showing, respectively, that anxiety is involved at every level of the interoceptive process, especially at its highest ones (Harrison et al., 2021a) and that the intriguing intra-subject differences in dyspnea for a given intensity of underlying mechanism (physiological changes) is positively related to anxiety sensitivity in healthy volunteers (Faull et al., 2017). The second alteration of the BPC process, i.e., imprecise physiological input, has been attributed to several factors including poor interoceptive abilities in scFS patients (Bogaerts et al., 2010; Van Den Houte et al., 2018) as well as, here again, in otherwise healthy high symptom reporters, expectation of imprecise physiological input, as well as to an increased activation of affective networks (Van den Bergh et al., 2017).

Most of the before-mentioned aspects of the altered BPC process may explain several of every-day clinical observations in DBD patients. Thus, the frequently observed expectation (and actual occurrence of) dyspnea, at exercise in DBD patient, fits well with heightened expectation of health problems and attribution of normal exercise-induced changes of physiological input to disease. Likewise, the underlying mechanisms of the second alteration of the BPC process, i.e., imprecise physiological input may explain several clinical observations in DBD patients, such as the so-called disproportional dyspnea, i.e., high intensity of dyspnea in case of low intensity physiological input may be related to the low precision of that input. It further may explain the *a priori* counterintuitive observation that anxious, hyper-vigilant and health and body-focused subjects are less accurate in their perceptual abilities. Likewise, this renewed view of symptom perception offers an explanation for the common clinical observation that symptom reports in DBD patients may be imprecise, sometimes even with an unreliable characterization of the symptoms (22% in our practice, unpublished results) that we initially attributed to the young age of the children attending our Dyspnea Clinic. Moreover, and most importantly, the BBH provides an alternative explanation for the intriguing dyspnea at rest in mostly otherwise healthy subjects which has been previously attributed to inappropriate cortical processing of respiratory-related sensory inputs (Jack et al., 2010) or to central sensitization (Bokov et al., 2016), that, moreover, is a still controversial concept (Van den Broeke et al., 2018).

More generally, it appears that, the degree of coupling between the ultimate consciously perceived symptom and its corresponding physiological inputs is highly variable according to the context and varies over time. Thus, each and every symptom lies somewhere along a continuum ranging from absence of, to a very tight relationship with every given putative physiological input. Consequently, symptoms of DBD, or more generally of scFS, are simply a special case of the great number of possible values of this continuum, i.e., on, or close to one of its extreme ends. Thus, perception in the case of DBD is not “high,” “inaccurate” or “bad” but only reflects the fact that the relative contribution of physiological input is low, or in Bayesian terms, its precision weight is low. Furthermore, the traditional differentiation between supposedly “explained” and “unexplained” symptoms appears to be inappropriate since all of them are actually explained by a unifying neuro-cognitive process. This renewed view further explains the intriguing, sometimes important, inter-and as well as the before-mentioned intra subject variability of symptom intensity for a given physiological input, a frequent clinical observation, notably for dyspnea. All in all, in the light of the BBH model, we clearly move away and transcend the previously supposed unique and stable one-to-one relationship between the perceived symptoms and physiological input.

#### 2.2.1. Implications of the BBH on assessment and management of DBD

A further contribution of the BBH is to open perspectives for new tests, i.e., specifically based on the assessment of the different operations of the BPC process, which is of clinical relevance given the previously mentioned limitations and difficulties regarding current characterization of DBD. While, due to the fact that the latter are predominantly unconscious, the assessment of some of them, especially priors, are not easily accessible to investigation, this issue has been partly circumvented during recent years by indirect approaches. Thus, the filter detection test (Harrison et al., 2021b), by a thorough assessment of the different aspects of the interoceptive process, i.e., accuracy and metacognitive awareness (Garfinkel et al., 2016) provides valuable insight into this crucial determinant of prediction error precision. A further test, the thermal grill illusion, a paradoxical heat-pain experience has also been previously used to investigate the relative contribution of priors and physiological input (Bräscher et al., 2020). Another indirect but important approach is that of the identification and assessment of personality traits or states that have previously been shown to be frequently associated with the crucial false inferences of the BPC process by use of specific questionnaires, e.g., Breathlessness Catastrophizing Scale (Solomon et al., 2015), the Fear of Suffocation subscale of the Claustrophobia Questionnaire (Radomsky et al., 2001), the Intolerance of Uncertainty Scale (IoU) (Helsen et al., 2013), the positive and negative affect scales (Watson et al., 1988), as well as the recently developed Breathing Vigilance Questionnaire (Steinmann et al., 2023). In contrast, given this low degree of coupling between symptoms and physiological input, as well as the previously mentioned non-specific nature of symptoms and putatively associated physiological dysfunction, it is somewhat unlikely that research primarily focused on identification of specific underlying physiological mechanisms will be very helpful for the characterization of DBD. This may however not be a crucial drawback since the specificity of this condition is the nature (strength) of the relationship between its symptom and physiological input, rather than physiological changes *per se*.

Regarding therapeutic management of DBD, the contribution of BBH to therapeutic management of DBD is twofold. First it offers a possible explanation of the way of action of current applied treatment options such as respiratory rehabilitation (Barker et al., 2016; Hepworth et al., 2019) and breathing exercises (Mahut et al., 2014;)^1^ which is sometimes attributed to a rather fuzzy concept of “reassurance” (Mahut et al., 2014;). As previously shown in a brain imaging study in COPD patients, the effect of the before-mentioned treatment strategies may act via a change (decrease) in learned association of contextual cues and in dyspnea-related anxiety (Herigstad et al., 2017). In addition, as suggested by the results of current practice in our pediatric Dyspnea clinic, explanations about exercise-physiology at the beginning and during of supervised exercise are able to suppress or at least, substantially decrease dyspnea in almost all children which may be related to rapid potentially temporary changes in conscious expectation and thereby prior formation by distraction. Second, and most importantly, the BBH opens perspectives for new innovative treatments that specifically target one or several components of the dysfunctional BPC process, i.e., formation of new priors, as for instance by disrupting associative learning by an effect on associative learning especially and improvement of interoceptive abilities by differentiation training (Schaefer et al., 2014; Meyerholz et al., 2019). New and promising contributing to a specific action on this altered PCB process may further come from drugs such as the—potentially somewhat difficult to handle—psychedelics, e.g., LSD and psilocybin, that induce a decrease in the precision of high-level priors, thereby increase the relative weight of physiological input (Carhart-Harris and Friston, 2019).

## 3. Concluding remarks

Thus, it turns out that the main characteristic of DBD is the way patients perceive their inner world and generate symptoms, which, themselves, are notoriously unspecific and variable and often common to, and indistinguishable from those of other mostly overlapping clinical conditions of the scFS nebula, suggesting that what’s important here is not *what* but rather *how* things are perceived. It may further be submitted that DBD is actually simply a respiratory expression among the many possible expressions of the scFS nebula. This does however not mean that DBD does not require and deserve both special assessment and treatment options that take into account its respiratory specificity and especially, that concerning its characteristic altered BPC process, therefore calling for a further development of the corresponding specific symptom-centered treatment options. In this respect, it is indeed worth remembering the predominant importance of symptoms as they are actually the expression of patients’ suffering and their motivation to seek medical help. This may be an incentive for focusing future research of DBD predominantly on the way its numerous symptoms are centrally processed rather than on unhelpful inherently disappointing temptations of defining diagnosis criteria and of classifications systems based on putative underlying physiological dysfunctions. Moreover, and most interestingly, the common view that symptoms of DBD are “in the head” remains true, but crucially, under a completely different perspective namely that to consider that this is a key characteristic of each and every consciously experienced symptom since all of them are cognitive constructs irrespective of the strength of their relation with sensory input.

In conclusion, even if one keeps in mind that the BBH is only a model, i.e., the best possible explanation at a given historical moment, it is undoubtfully a major step forwards in our understanding of DBD and its numerous related symptoms, thereby contributing to improve our management of this complex and distressing clinical condition.

## Data availability statement

The original contributions presented in the study are included in the article/supplementary material, further inquiries can be directed to the corresponding author.

## Author contributions

CP: Conceptualization, Writing – review & editing.

## Footnotes

^1^Coquelin, F., Bourmaud, A., Fuchs, A., Toumazi, A., Coste, P., Pesle, A., et al. (under review) Assessing the impact of the Robert Debré pediatric dyspnea clinic management on patients with unexplained exertional dyspnea. Pediatr. Pulmonol.
